# A novel algorithm for initial lesion detection in ultrasound breast images

**DOI:** 10.1120/jacmp.v9i4.2741

**Published:** 2008-11-11

**Authors:** Moi Hoon Yap, Eran A. Edirisinghe, Helmut E. Bez

**Affiliations:** ^1^ Department of Computer Science Loughborough University Loughborough U.K.

**Keywords:** medical image analysis, ultrasound imaging, region‐of‐interest, hybrid filtering, multifractals

## Abstract

This paper proposes a novel approach to initial lesion detection in ultrasound breast images. The objective is to automate the manual process of region of interest (ROI) labeling in computer‐aided diagnosis (CAD). We propose the use of hybrid filtering, multifractal processing, and thresholding segmentation in initial lesion detection and automated ROI labeling. We used 360 ultrasound breast images to evaluate the performance of the proposed approach. Images were preprocessed using histogram equalization before hybrid filtering and multifractal analysis were conducted. Subsequently, thresholding segmentation was applied on the image. Finally, the initial lesions are detected using a rule‐based approach. The accuracy of the automated ROI labeling was measured as an overlap of 0.4 with the lesion outline as compared with lesions labeled by an expert radiologist. We compared the performance of the proposed method with that of three state‐of‐the‐art methods, namely, the radial gradient index filtering technique, the local mean technique, and the fractal dimension technique. We conclude that the proposed method is more accurate and performs more effectively than do the benchmark algorithms considered.

PACS numbers: 87.57.Nk

## I. INTRODUCTION AND SCOPE

Breast cancer is the most common of all cancers affecting women in the developed countries.^(1)^ More middle‐age women die of breast cancer than of any other single cause.^(1)^ In the United Kingdom, more than 41 000 cases are diagnosed annually, and it is predicted that 1 in every 9 women will develop breast cancer at some point in life.^(2)^ Early detection plays a significant role in the fatality of breast cancer. Technologies that aid in the early detection of cancers have therefore attracted much attention from the research community.

Mammography and ultrasound imaging are the standard technologies used in cancer screening. Mammography is accepted as the “gold standard” for breast imaging. It is widely used as the primary tool for cancer screening. However, in diagnostic workup, mammography and breast ultrasound are often used as complementary investigations. Mammography has been shown to cause high false‐positive rates in diagnosis, and the radiation dose to the breast is harmful.^(3)^ Further, cost considerations have resulted in most countries choosing to use screen film mammography instead of a digitized version. However, the inability to change image contrast or brightness, problems in detecting subtle soft‐tissue lesions (dense glandular tissues), and difficulties with archiving have limited the application of screen film mammography.

Ultrasound studies have been shown to be good at picking up many of the cancers missed by mammography, especially in women who have dense breasts. In addition, ultrasound is noninvasive, portable, and versatile. Further, it does not use ionizing radiation, and it is relatively lower in cost. However, ultrasound images have a major disadvantage: poor quality because of multiplicative speckle noise that results in artifacts. Segmentation of lesions in ultrasound images is therefore a challenging task that remains an open problem despite many past research efforts.

In most existing breast screening approaches, the “initial lesion”—that is, a suspect region—is manually located by a trained radiologist in a pre‐processing stage by marking its topmost, leftmost, bottommost, and rightmost boundary limits with crosses. These crosses (and hence the initial lesion) are then manually encompassed within a rectangular region of interest (ROI)^(4)^ by the radiologist and subsequently presented to a computer‐aided diagnosis (CAD) system for further analysis leading to the segmentation and classification of the tumor. Because the selection of extreme points (that is, the crosses) and the rectangular region both require human intervention, these steps are open to subjectivity and human error. As a result, a well‐trained and experienced examiner with knowledge of the normal echo anatomy of the breast and the changes caused by pathology is required for accurate breast cancer screening. A failure to include an entire lesion within the ROI or to outline ROIs containing lesions (among other errors) can severely undermine the performance of the CAD system. Further, it has also been shown that radiologists with varying training backgrounds and experiences often reach rather different results in the reading of sonograms.^(5)^

Our current research focus is to provide the radiologist with an automated tool that can effectively assist in the selection of the ROI and in improving the consistency of interpretation. However, it is worthwhile noting that the automatic detection of ROIs is not meant to replace the radiologist, but to provide a tool to reduce the radiologist's ROI labeling time (see Section II) and to warn of possible ROIs that might otherwise be missed because of the poor quality of the ultrasound image.

Evaluation of the performance of the proposed algorithm requires a suitable test image database, a suitable evaluation metric, and a design goal. Because of the practical difficulties in obtaining databases with ultrasound images of normal and nearly normal breasts, previous state‐of‐the art algorithms^(6)^ ^,^ ^(7)^ of initial lesion detection have used U.S. image databases that consist solely of malignant and benign tumors. In our experiments, we used a U. S. image database of 360 malignant and nonmalignant images that additionally contains location information concerning the extreme points of tumors (marked with crosses by a number of expert radiologists). The metric used to evaluate the performance of the algorithm on the database is an “overlap” figure^(6)^ defined as the ratio of the intersection and the union of the two lesion areas that were manually identified by the radiologists and by the computer‐based algorithm. Specifically, in work by Drukker et al.^(6)^ and Yap et al.,^(7)^ the design goal has been to achieve an overlap value of in excess of 0.4, which can be represented as

(1) $$overlap=\frac{X\cap Y}{X\cup Y}\geq 0.4,$$

where *X* is the lesion area extracted by the computer‐based algorithm, and *Y* is the lesion labeled manually by the radiologists. In other words, the assumption has been that, if an overlap of 0.4 or more results, then the computer‐based algorithm has been successful in accurately and automatically performing the otherwise manual task of initial lesion identification. A secondary metric, “accuracy,” can therefore be defined as the percentage of experiments obtaining an overlap value of beyond 0.4. In the present research context, we use “accuracy” as the objective metric to evaluate and compare the results of the performance of the proposed algorithm with that of state‐of‐the‐art methods. Further subjective results have been illustrated for visual comparison.

The present paper is divided into five sections for clarity of presentation. Section II discusses our research motivation and the existing solutions to the problem. Section III introduces the proposed approach and provides a detailed discussion of each stage. Section IV sets out the experimental results and discusses the results in detail. Finally, Section 5 concludes with further directions for improvement and research.

## II. MOTIVATION AND RESEARCH BACKGROUND

**Figure 1:**
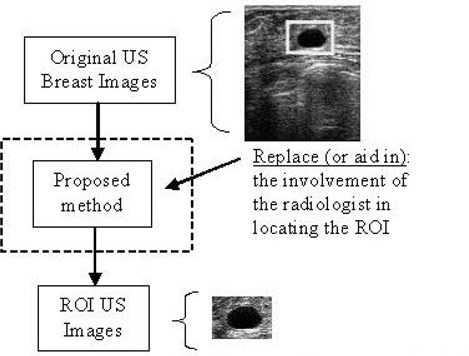
Use of the proposed solution. US=ultrasound; ROI=region of interest.

Many ongoing ultrasound breast imaging research projects are focused on creating CAD tools that have high sensitivity, specificity, and consistency in lesion classification. Examples include Boone,^(8)^ Gurney,^(9)^ Boukerroui et al.,^(10)^ Chen et al.,^(5)^ and Sehgal et al.^(11)^ Unfortunately, these systems are based on the assumption that the ROI will be pre‐selected by a radiologist and that the analysis will be performed only on the cropped ROI. This requirement improves accuracy in detecting lesion shape because the noisy, dark, poor‐quality surrounding areas can be excluded from consideration because of the manual selection of the specific ROI. It therefore follows that a fully automated CAD tool used in cancer screening will require a preprocessing stage that is capable of automatic ROI labeling. Our work is focused on providing an effective solution to that problem (see Fig. 1).

Two existing state‐of‐the‐art techniques for automatic ROI labeling of existing breast images, used as benchmarks to compare the performance of the proposed technique, can be summarized as follows:

- Drukker et al.^(6)^ investigated the use of the radial gradient index (RGI) filtering technique to automatically detect lesions on breast ultrasound images. In ultrasound images, lesions are almost invariably darker than the background. Thus, in Drukker's work, the grayscale of the original ultrasound images is initially inverted. Subsequently, images are pre‐processed by a median filter to remove speckle noise. The resulting image is fed to a RGI filter.^(6)^ In RGI filtering, the images are sub‐sampled by a factor of 4. A brightness threshold (see Subsection III.E) for the RGI‐filtered image is varied iteratively from 0.74 to 0.62 until either at least one lesion of interest is detected. The detected areas smaller than $5\;{\text{mm}}^{2}$ are discarded. Lesion candidates are segmented from the background by maximizing an average radial gradient (ARD) index for regions grown from the detected points. According to Drukker et al., maximizing the ARD index is more accurate than maximizing the RGI index.^(6)^ At an overlap level of 0.4 with lesions outlined by a radiologist, 75% accuracy of lesion detection was reported for the test set of ultrasound images used.
- Yap et al.^(7)^ analyzed the use of statistical methods (for example, local mean) and values of fractal dimensions in initial lesion detection. The images were preprocessed using histogram equalization,^(12)^ and then hybrid filtering (see Subsection III.C) and marker‐controlled watershed segmentation were applied. (A “watershed” is the ridge that divides areas drained by different river systems. A catchments basin is the geographic area draining into a river or reservoir. The watershed transform applies these ideas to grayscale image processing in a way that can be used to solve a variety of image segmentation problems.) Marker‐controlled watershed segmentation is an approach based on the concept of markers to control oversegmentation in watershed transform.^(13)^ The minimum local mean and the minimum of the fractal dimensions (see Subsection III.D) of the identified segments were then used to identify the initial lesion. Subsequently, neighborhood segments are identified, and these are finally combined to form the ROI. The accuracy of the automated ROI labeling is measured by an overlap of 0.4 between its lesion outline and the lesions labeled by the radiologists. The accuracy of ROI detection when using local mean was reported to be 69.21%; fractal dimension was 54.21%.

The proposed approach detailed in the next section intends to further extend the accuracy of ROI detection by following an effective multistage algorithm.

## III. PROPOSED APPROACH

**Figure 2:**
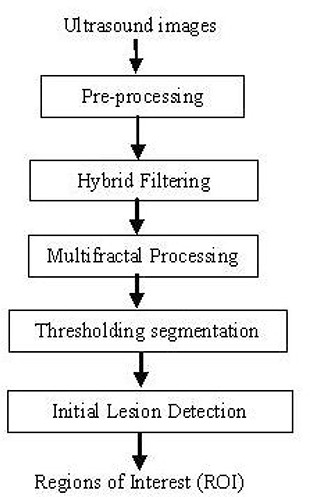
Overview of the methodology.

Fig. 2 shows a modular block diagram of the proposed technique. It uniquely combines histogram equalization as a preprocessing stage, followed by hybrid filtering, multifractal analysis, thresholding segmentation, and a rule‐based approach in fully automated ROI labeling.

The operation and functionality of the individual stages are described in detail in the subsections that follow. All algorithms were implemented in LTI‐Lib^(14)^ on a Linux platform.

### A. Ultrasound images

In general, ultrasound images are complex because of data composition, which can be described in terms of speckle information. Upon visual inspection, speckle noise consists of a relatively high‐level gray intensity, qualitatively ranging between hyperechoic (bright) and hypoechoic (dark) domains.^(15)^

Notably, any automatic system that is designed to detect abnormal lesions in ultrasound images should, in the end, be verified or compared with the judgment of a medical expert or radiologist. The test images used in our work were obtained from a professionally compiled compact disc (CD) of breast ultrasound images,^(16)^ which consists of explanations and verifications from several qualified expert radiologists. We selected 360 images from the CD for our experiments. Of the 360 images, 20 were malignant, 76 were simple cysts, 76 were complex cysts, 58 were fibroadenomas, 38 were carcinomas, 18 were occult lesions, 15 were adenosis lesions, and 59 were a combination of other diagnoses. Each image had been manually processed by an expert radiologist, and the extreme points of the suspected lesions were already marked with crosses. Because the objective metric of the experiment (see Section I) required identification of the lesion boundary marked by a radiologist (rather than the extreme points or ROI), we obtained the services of an expert radiologist to mark these boundaries manually and to verify the extreme points given in the ultrasound CD.^(16)^

### B. Preprocessing

As mentioned previously, the credibility of a high‐quality breast ultrasound examination depends on the scanner (that is, the quality of the original image) and the experience of the examiner. The preprocessing stage deals with the issue of guaranteeing the homogeneity of the original ultrasound images, which thus subsequently improves the chances of lesion ROI detection being more accurate. In the proposed approach, we use a histogram equalization strategy tested in earlier experiments^(17)^ as a preprocessing stage to achieve the homogeneity guarantee.

Histogram equalization^(12)^ is similar to contrast stretching, in that it attempts to increase the dynamic range of the pixel values in an image. However, unlike contrast stretching, no provision is made for interactivity, because applying a histogram equalization algorithm to an image with a fixed number of bins will always yield the same result. Let

(2) $$p_{r}(r_{j}),j=1,2,\ldots ,L$$

denote the histogram associated with the intensity levels of a given image, and recall that the values in a normalized histogram are approximations to the probability of occurrence of each intensity level in the image. For discrete quantities, the equalization transformation becomes

(3) $$s_{k}=T(r_{k})=\sum_{j=1}^{k}p_{r}(r_{j})=\sum_{j=1}^{k}\frac{n_{j}}{n}$$

for k=1,2,…,L, where $s_{k}$ is the intensity value in the output (processed) image corresponding to value $r_{k}$ in the input image, *n* is the total number of pixels, and $n_{j}$ is the number of pixels in bin *j*.

### C. Hybrid filtering

The function of the filtering stage is to remove noise, which is a major obstacle to accurate segmentation of the images. Median filtering is a popular approach used in removing speckle noise in ultrasound images.^(6)^ ^,^ ^(18)^ ^,^ ^(19)^ However, Yap et al.^(17)^ proved that part of the reason for the inaccuracy of the boundary detection in Drukker et al.,^(6)^ Joo et al.,^(18)^ and Kupinski et al.^(19)^ was that, although the median filter managed to filter out the speckle noise, it also removed the important edge information—in particular, edges that belonged to the lesion.

Further, Gaussian blur^(4)^ is a linear filtering technique that has been widely used to reduce the oversegmentation problem in ultrasound images. Gaussian blur is very effective in removing speckle noise, but it blurs and dislocates edges,^(20)^ which may negatively affect subsequent lesion segmentation. Perona and Malik^(21)^ proposed a nonlinear partial differential equation approach for smoothing images on a continuous domain. Anisotropic diffusion was shown to perform well for images corrupted by additive noise. However, in cases where images contain speckle noise, anisotropic diffusion enhances that noise instead of eliminating it.^(22)^ On a more positive note, nonlinear diffusion filtering.^(23)^ as compared with linear diffusion, has deservedly attracted much attention in the field of image processing for its ability to reduce noise while preserving (or even enhancing) important features of the image such as edges or discontinuities.

**Figure 3:**
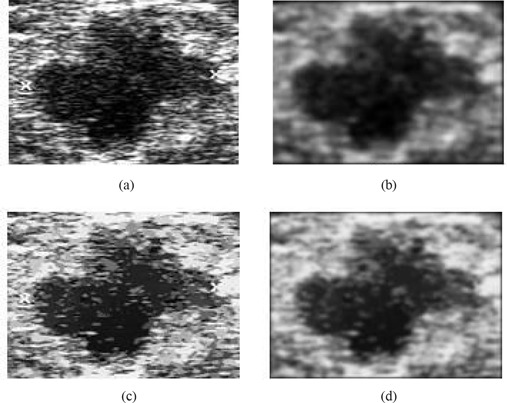
Comparison of filtering approaches: (a) original image; (b) Gaussian blur; (c) nonlinear diffusion filtering; (d) hybrid filtering.

Within the context of proposed research, we make use of a hybrid filtering approach that combines the strength of nonlinear diffusion filtering to produce edge‐sensitive speckle reduction, with linear filtering (Gaussian blur) to smooth the edges and to eliminate oversegmentation. The result of hybrid filtering is visually compared in Fig. 3. Section IV sets out our experimental results and a detailed analysis to justify the use of hybrid filtering as compared with either nonlinear diffusion filtering or Gaussian blur alone.

Subsequent to hybrid filtering, we use multifractals^(24)^ to further enhance the partially processed images. Section IV sets out experimental results to show that this stage allows for better segmentation of lesions than does the application of hybrid filtering only. Subsection D, next, provides a brief overview of multifractals and associated analysis methodology.

### D. Multifractal dimensions

A fractal is generally “a rough or fragmented geometric shape that can be subdivided in parts, each of which is (at least approximately) a reduced‐size copy of the whole,”^(25)^ a property called self‐similarity. In analyzing the fractal geometry of an image, an attempt is made to exploit the self‐similarity present. In fractal geometry, the term “fractal dimension” refers to a statistical quantity that indicates how completely a fractal appears to fill space, as one zooms down to finer and finer scales.^(25)^ Multifractal analysis refers to the analysis of an image using multiple fractals—that is, not just one fractal, as in fractal analysis.

The generalized formulation for multifractal dimensions *D* of order *q* ^(24)^ can be represented as

(4) $$D_{q}=\left\{\begin{array}{lll} \frac{1}{q-1}\underset{\varepsilon \to 0}{\lim}\frac{\log(x_{q}(\varepsilon ))}{\log(\varepsilon )} & & for\;q\in ℜ\;and\;q\neq 1\\ \underset{\varepsilon \to 0}{\lim}\frac{\sum_{i}\mu_{i}log\mu_{i}}{\log(\varepsilon )} & & \;\;forq=1 \end{array}\right.,$$

where ∊ is the linear size of the cells (in our case, because we use a 3×3‐pixel mask, ɛ=3), and *q* is the order for cell size ∊. Note that when $q<0,\;D_{q}$ is sensitive to the parts where the measure is very dense. On the other hand, if q>0, information on the sparse region can be obtained. In theory, *q* is in the range –∞ to ∞, and $D_{g}$ can have an infinite number of values. In practice, computing for all values of *q* is not possible. Hence, we empirically decided to use only four values of *q* in our experiments: −1,0, 1, and 2.

The partition function $\chi_{q}$ is defined as

(5) $$x_{q}(\varepsilon )=\sum_{i=1}^{N(\varepsilon )}\mu_{i}^{q}(\varepsilon ),$$

where *N*(∊) is the total number of cells of size ∊, and $\mu_{i}^{\theta }(ɛ)$ is the measure that is defined on a given set. In this case, the measures are defined as the probability of the grayscale level in the images, where all the gray levels fall in the range of 0 – 1.

To investigate the effect of various *q* values on ultrasound images, we carried out further empirical experiments.

**Figure 4:**
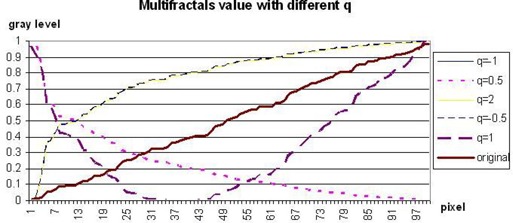
Graph of multifractals dimension with various values of *q*.

**Figure 5:**
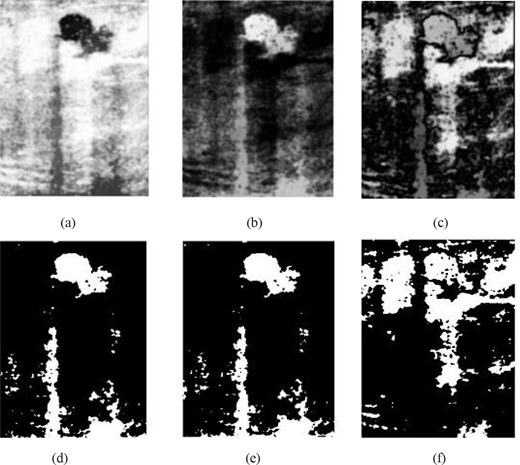
Result of multifractal analysis with (a) q<0 or q>1; (b) 0<q<1; (c) q=1; (d) threshold of (a); (e) threshold of (b); and (f) threshold of (c).

The graph in Fig. 4 shows that the multifractals with −∞<q<∞ yields four types of results. We can classify those results into q=0, q=1, 0<q<1, and (q<0 or q>1). Note that 0<q<1 is the inverted value of (q<0 or q>1). From the results illustrated in Fig. 5, it is seen that any value range 0<q<1 or (q<0 or q>1) will help improve the segmentation results. The value q=−1 (that is, $D_{-1})$ is chosen in our work because, as compared with other values, it has the lowest associated computational complexity.

To our knowledge, hybrid filtering has not been used in previous research in breast ultrasound boundary detection or contrast enhancement, and hence its use is an additional novel aspect of our approach.

After application of the hybrid and multifractal filtering, the images are ready for lesion segmentation. Subsection E introduces the approach adopted.

### E. Thresholding segmentation

In general, segmentation is a process used to divide an image into its constituent parts. Thresholding segmentation^(26)^ is the most basic, the simplest, and the fastest algorithm in segmentation. A thresholding procedure attempts to determine an intensity value, called the “threshold,” that separates pixels into desirable classes. A parameter θ, called the “brightness threshold” is chosen and applied to the image *a*[*m*, *n*] as follows:

(6) $$\begin{array}{ll} & If\;a[m,n]\;{}^{3}q\\ & then\;a[m.n]:=1(object)\\ & else\;a[m.n]:=0(background) \end{array}$$

Within our present research context, we used a fixed threshold for segmentation—that is, a threshold chosen independently of the image data. If it is known that very high‐contrast images (where the objects are very dark and the background is homogeneous and very light) are being dealt with, then a constant threshold of 128 on a scale of 0 to 255 was experimentally found to be sufficiently accurate. That is, the number of falsely‐classified pixels is kept to a minimum. The sensitivity of the threshold selection on segmentation accuracy was further found to be low, justifying the use of a image‐independent fixed threshold.

### F. Initial lesion detection

The thresholding segmentation of Subsection III.E often leads to the identification of multiple ROIs, of which generally only one or two would be of diagnostic importance (that is, belonging to abnormal lesions). Further, the location of the abnormal lesions requires the specification of both position and orientation (assuming two‐dimensional ultrasound images). We therefore propose the use of a rule‐based approach to identify these important ROIs.

The first criterion for the identification of lesions is the size of the segments. The suspect lesions are identified as the largest segments among the likely multiple segments that result from applying the single‐threshold segmentation. In addition, based on the additional guidance provided in the ultrasound image CD from which we obtained our test data,^(15)^ we observed that 95% of tumors are located at the upper regions of the images. Hence, a reference point at (*x*, *y*), where

(7) $$x=\frac{image\;height}{3},and\;y=\frac{image\;width}{2}$$

is chosen as the center of attention. The lesion that is located closest to that point and that satisfies the above mentioned size‐related criterion is selected as the final detected lesion. The size‐related criterion can be appropriately slightly relaxed if more than one lesion must be detected.

## IV. RESULTS AND ANALYSIS

**Figure 6:**
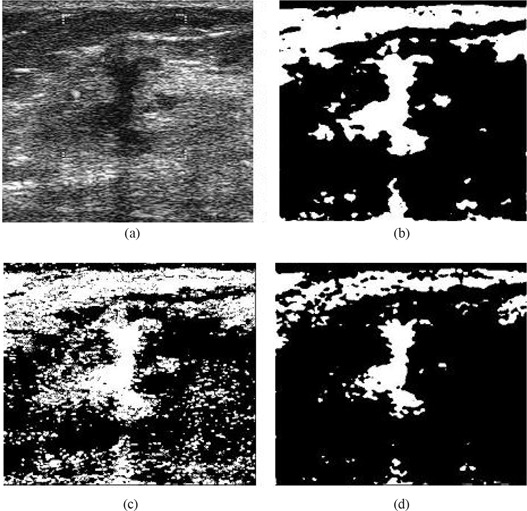
Comparison of segmentation approaches: (a) original image, and use of (b) Gaussian plus multifractal filtering, (c) nonlinear plus multifractal filtering, (d) hybrid plus multifractal filtering.

Fig. 6 compares the effects of using the various filtering techniques discussed in Section III with multifractal processing. When Gaussian filtering is used [see Fig. 6(b)], the smoothing effects introduced at the edges result in noisy regions that ideally should be disconnected remaining connected. This effect is a problem in the subsequent lesion detection stage, because the largest connected region may not now refer to the true lesion that should be detected. The use of nonlinear filtering results in oversegmentation and causes many problems, as illustrated by Fig. 6(c). However, with the use of hybrid filtering and multifractal processing, the single largest segmented area detected is identified as the lesion.

**Figure 7:**
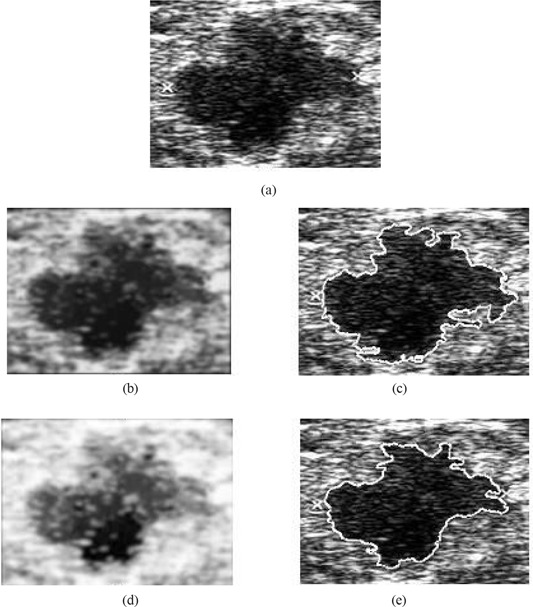
Justification of boundary enhancement with multifractal processing. (a) Original image; (b) hybrid filtered image; (c) boundary detected on hybrid filtered image; (d) multifractal processed image; (e) boundary detected on multifractal processed image.

Fig. 7 compares final lesion boundary detection accuracies for the use of hybrid filtering alone and for hybrid filtering followed by multifractal processing. The boundary detection accuracy in Fig. 7(e) is better than that in Fig. 7(c), justifying the positive contribution of multifractal processing to subsequent boundary detection. The boundary in Fig. 7(c) is closer to the boundary that a typical radiologist might intend to identify, because the boundary is smoother and excludes effects of noise and other artifacts more effectively.

**Figure 8:**
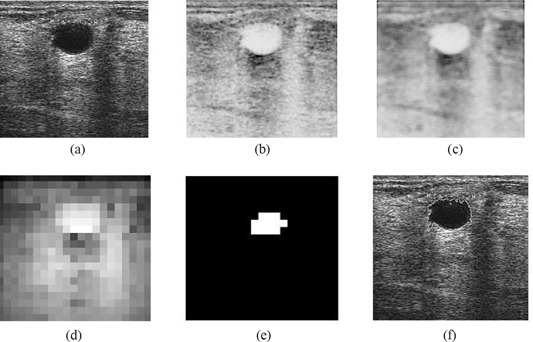
Illustration of the operation of the intermediate stages of the Drukker et al.^(6)^ algorithm. (a) Original image; (b) grayscale inverted image; (c) median filtered image; (d) radial gradient index (RGI) filtered image; (e) thresholded RGI image; (f) final detection.

**Figure 9:**
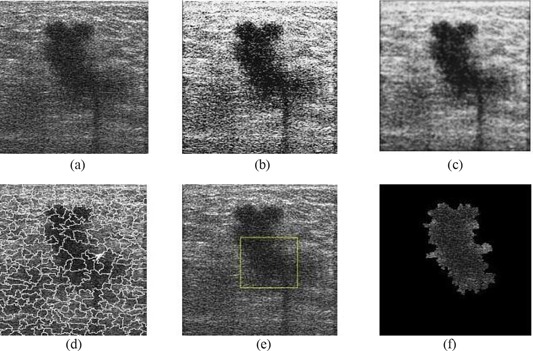
Illustration of the operation of the intermediate stages of the Yap et al.^(7)^ algorithm (local mean). (a) Original image; (b) image after preprocessing (histogram equalization); (c) image after hybrid filtering; (d) watershed segmentation mapped onto the original image; (e) location of the initial lesion; (f) combination of the neighborhood segments with the initial lesion.

**Figure 10:**
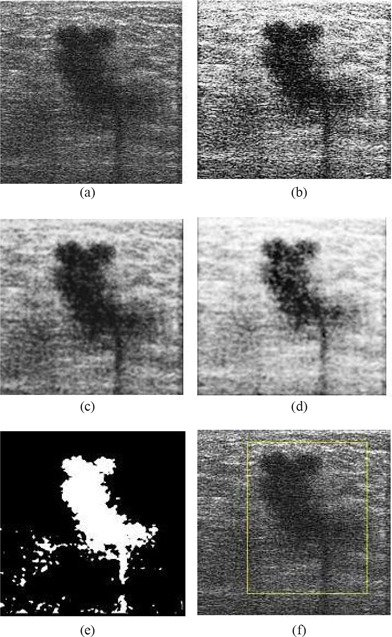
Illustration of the operation of the intermediate stages of the proposed algorithm. (a) Original image; (b) image after pre‐processing (histogram equalization); (c) image after hybrid filtering; (d) image after multifractal processing; (e) image after thresholding segmentation; (f) labeling of region of interest.

Figs. 8, 9, and 10 illustrate the stage‐by‐stage operation of the algorithms from Drukker et al.,^(6)^ Yap et al.,^(7)^ and the present proposal. These three algorithms use different steps in identifying lesions. The intentions at each stage within the three algorithms are significantly different, and hence, the intermediate results are not comparable. A more comprehensive performance comparison based on the accuracy of the final detected lesion regions is given later in the present paper.

**Figure 11:**
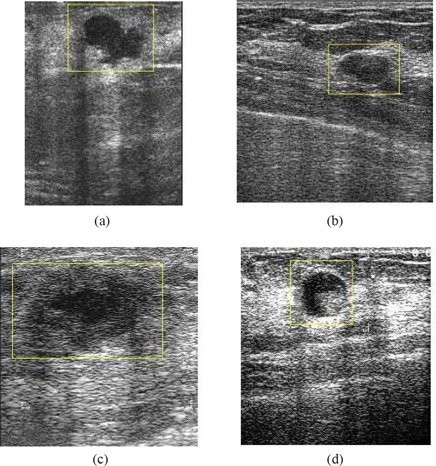
Results of automated region of interest lesion labeling using the proposed method. (a) Malignant tumour; (b) simple cyst; (c) fibroadenoma; (d) complex cyst.

**Figure 12:**
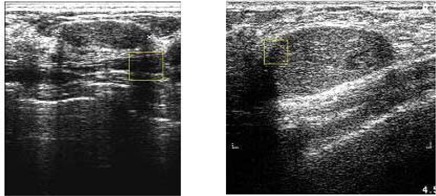
Examples of unsuccessful lesion identification for two cases of fibroadenoma.

Our detailed experiments, performed using the 360 test ultrasound images, revealed that the proposed method performs exceptionally well in identifying ROIs for most cyst lesions and malignant lesions, and for some fibroadenoma lesions. Because of the high degree of similarity in texture between normal and fibroadenoma regions, accurate identification of such regions is always a challenge. Fig. 11 provides a visual comparison of the results of using the proposed algorithms on various types of abnormalities. Fig. 12 illustrates two examples of fibroadenoma ROIs that are not detected accurately.

**Figure 13:**
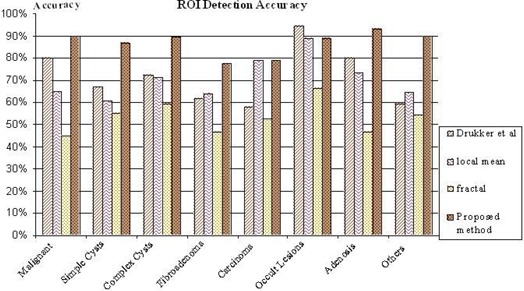
Graphical presentation of the region of interest (ROI) detection accuracy for each of the four algorithms and each type of abnormality.

Based on diagnoses by radiologists as provided on the test ultrasound breast imaging CD used in our experiments,^(16)^ Table 1 summarizes the accuracy figures obtained for each type of abnormality for the three benchmark algorithms and the proposed algorithm. Fig. 13 graphically presents ROI detection accuracy for each of the four algorithms and each type of abnormality. The results clearly show the improvements obtainable with the proposed improved approach to ROI lesion detection accuracy. The detection accuracy for fibroadenoma‐type lesions has generally been the lowest for all methods, but in this category, the proposed algorithm shows a 15% accuracy improvement as compared with the methods of Drukker et al.^(6)^ and Yap et al.^(7)^ Further, when using the proposed algorithm, 90% accuracy in detecting malignant‐type lesions is indicated.

**Table 1:** Summary of region of interest detection accuracy for each type of abnormality under four different algorithms

*Diagnosis*	*Total images (*n*)*	*Drukker et al*.^(6)^	*Accuracy (%)*		
			*Yap et al. (local mean)* ^(7)^	*Yap et al. (fractal)* ^(7)^	*Proposed method*
Malignant	20	80.00	65.00	45.00	90.00
Simple cysts	76	67.11	60.53	55.26	86.84
Complex cysts	76	72.37	71.05	59.21	89.47
Fibroadenoma	58	62.07	63.79	46.55	77.59
Carcinoma	38	57.89	78.95	52.63	78.95
Occult lesions	18	94.44	88.89	66.67	88.89
Adenosis	15	80.00	73.33	46.67	93.33
Others	59	59.32	64.41	54.24	89.83
Total	360	67.78	68.05	53.89	86.11

**Figure 14:**
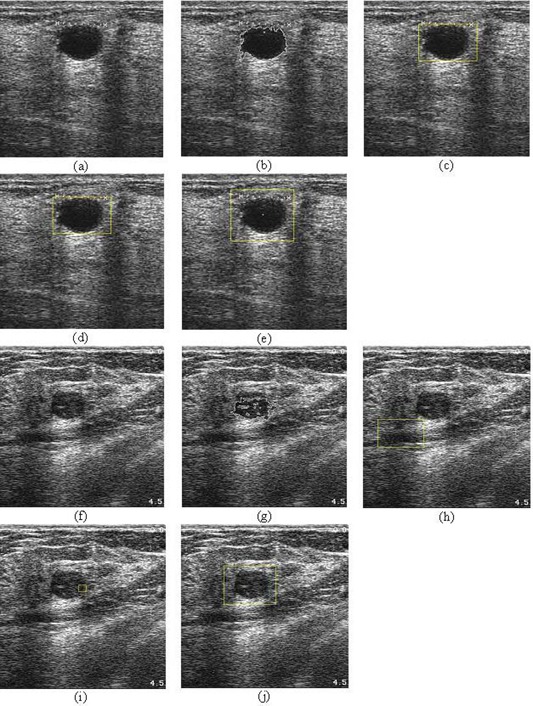
Visual performance comparison with the benchmark algorithms. (a,f) Original images; (b,g) results of the Drukker et al.^(6)^ algorithm; (c,h) results of the Yap et al.^(7)^ algorithm (local mean); (d,i) results of the Yap et al.^(7)^ algorithm (fractal dimension); (e,j) results of the proposed method.

Fig. 14 visually compares the performance of the proposed approach with that of the three benchmark algorithms. This comparison clearly illustrates the improved accuracy of lesion identification demonstrated by the proposed approach. The three benchmark algorithms can be seen to be more likely to identify non‐lesion regions—or only parts of lesions—as ROIs.

## V. SUMMARY AND CONCLUSIONS

We propose a method that is able to fully automate ultrasound CAD. We have successfully proved that the proposed algorithm achieves an improvement as compared with benchmark algorithms. The proposed method is able to very accurately label most lesions, with its best performance being the identification of malignant lesions (90%) and its worst being the identification of fibroadenomas (77.59%). We are currently considering the use of shape information and frequency domain analysis, among other techniques, to further improve the performance of the approach presented here.

A complete computer‐aided ultrasound diagnostic system (ultrasound CAD) must be able to classify the diagnosis of each ROI. In future, we will investigate the use of classification techniques such as neural networks and support vector machines, among others, to form a fully automated breast cancer detection system.
